# Mapping Metabolomic Relationships of Hop Cultivars in an Ancestral Lineage Context

**DOI:** 10.1021/acsomega.5c06162

**Published:** 2025-09-29

**Authors:** Guilherme Silva Dias, Marília Elias Gallon, Leonardo Gobbo-Neto

**Affiliations:** Department of BioMolecular Sciences, School of Pharmaceutical Sciences of Ribeirão Preto, 28133 University of São Paulo (USP), Av. do Café s/n, Ribeirão Preto, SP 14040-903, Brazil

## Abstract

Hops (*Humulus lupulus*) are one of the most valuable raw materials in the global brewing industry as they provide resins and essential oils highly relevant to beer production. Hop cultivars commonly used in brewing are predominantly derived from European and American genetic lineages, whose agronomic and phytochemical traits are frequently studied to support the development of new varieties increasingly adapted for beer production. Herein, we investigated the chemical profiles of 76 different commercial hop cultivars using gas chromatography coupled to mass spectrometry (GC-MS) analysis and a metabolomic approach. Unsupervised statistical analyses revealed a clustering tendency between cultivars of North American or European lineages. Chemical composition exploration was conducted through molecular networking, allowing the annotation of 26 metabolites that distinguished the two groups. American lineage hops exhibited a chemical profile characterized by mono- and sesquiterpenoids, ketones, and esters, whereas hops from European lineage presented higher abundances of α- and β-selinene, *trans-*α-bergamotene, humulene epoxide II, neophytadiene, and tocopherols. Our findings strengthen the evidence for selinenes as potential markers for differentiating hops of European genetic background and highlight *trans-*α-bergamotene as an important chemical marker in hop cultivars. Thus, GC-MS-based metabolomic signatures of multiple commercial hop cultivars revealed distinguished chemical relationships of kinship among American and European lineages, advancing our understanding of cultivar chemical diversity in an ancestral lineage context and providing a metabolomic basis for hop cultivar classification and traceability.

## Graphical abstract

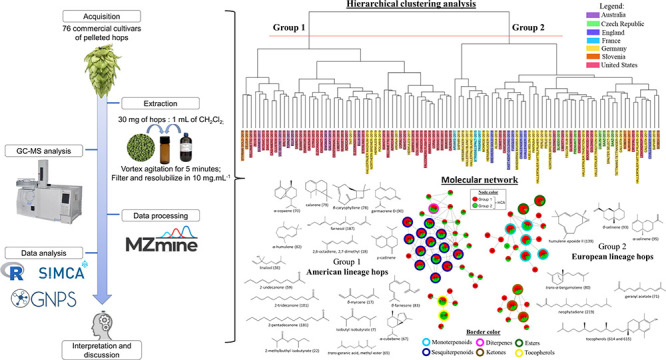

## 1 Introduction

Highly valued in the global brewing industry, hops are perennial, dioecious, herbaceous, liana-like plants that belong to the Rosales order and to the Cannabaceae family.^1,2^ The genus *Humulus*, which consists of three species—*Humulus lupulus* L. (*H. lupulus*, common hops), *Humulus japonicus* Siebold & Zucc., and *Humulus yunnanensis* Hu— is believed to have originated in China, although wild populations of *H. lupulus* are known to be widely distributed across the Northern Hemisphere.^3,4^ Accordingly, the history of hops along with their domestication and use in brewing remains surrounded by uncertainties.^1,4,5^

*Humulus lupulus* is the most economically and culturally significant species within the genus, having been used in beer production for centuries.^3^ Initial hop cultivars were derived through the selective breeding of wild European populations.^6,7^ A general agreement posits that North American and European wild varieties diverged from a common ancestor more than a million years ago.^4,8^ Currently, five geographically and morphologically distinct botanical varieties of *H. lupulus* are recognized: var. *humulus* (Europe and Asia), var. *cordifolius* (East Asia, particularly Japan), var. *neomexicanus* (southwestern North America), var. *pubescens* (eastern and north-central USA), and var. *lupuloides* (northeastern North America and Canada).^5,9^ Over time, cultivar development has evolved from open-pollinated crosses to controlled hybridization, incorporating North American germplasm and thus broadening the genetic base.^7^

The importance of hop cultivation is highlighted by global production, which ranges from 80,000 to 130,000 tons annually, with Germany and the United States being the world’s largest producers.^10,11^ The increasing demand for aromatic hops, driven by the American craft beer revolution dating back to the 1980s, has accelerated the development of new cultivars. Currently, approximately 300 commercial cultivars are available in the brewing market.^12–14^ Beyond imparting bitterness, flavor, and aroma, hops also exhibit antimicrobial and antioxidant properties, playing a crucial role in the organoleptic stability of beer.^15–17^

The main compounds in hops that are valuable for beer production are biosynthesized and accumulated in the inflorescences (strobiles, also referred as cones) of female plants, specifically in the glandular trichomes (lupulin glands) located at the bases of the bracteoles of the cones.^2,3^ The resins contain compounds and precursors that contribute to the bitterness of beer (e.g., α-acids and β-acids), while the essential oils are the main contributors to the aromatic profile, especially in beer styles that emphasize hop character.^18^ The amount of essential oils in hops can range from 0.5 to 4.0% of the dry cone weight and is primarily composed of terpenes (up to 90% of the essential oil), aliphatic hydrocarbons, oxygenated compounds (such as terpenoid, ester, ketone, alcohol, carboxylic acid, aldehyde, and epoxide derivatives), and organosulfur compounds.^19–21^

Analytical approaches such as gas chromatography coupled to mass spectrometry (GC-MS), liquid chromatography coupled to ultraviolet detectors and/or mass spectrometry (LC-UV-MS), and nuclear magnetic resonance (NMR) have been widely employed for hop characterization.^22–26^ For aroma profiling and quality control, GC-MS techniques are employed to analyze volatile compounds, whereas LC-UV-MS techniques are used to characterize bitter acids, polyphenols, and flavonoids.^22^ Volatile and semivolatile metabolites in hops are largely determined by genetic factors and, therefore, define characteristic phytochemical profiles for each hop cultivar.^2,27–29^ In this regard, a comprehensive GC-MS approach encompassing a wide variety of metabolites is essential for capturing lineage-associated differences in metabolite composition that may be overlooked by traditional workflows focusing solely on volatile compounds.^21,30^

Metabolomic approaches are powerful tools for investigating the chemotaxonomic diversity, including that of hop cultivars from various geographical origins.^26,31–33^ Despite the commercial significance of hop cultivars, comprehensive studies utilizing metabolomic techniques to analyze a broad spectrum of commercial varieties remain scarce. Given that beer quality is intrinsically linked to the chemical composition of its raw materials and ingredients (malted barley, hops, yeast, and water), comprehensive analyses of hop metabolites are helpful in selecting the best varieties to produce beers with desirable flavor profiles, balanced bitterness, and optimal aroma intensity. In this context, such metabolomic knowledge may provide valuable support for the brewing industry in cultivar selection, quality standardization, and product development. In this study, 76 hop cultivars, marketed in a pelletized form, were analyzed by using GC-MS metabolomic profiling. The resulting data were subjected to multivariate statistical analyses and molecular network techniques to identify chemotaxonomic patterns linked to the cultivars’ geographical origins. These findings provided novel insights into the chemical diversity of hops and offered frameworks for mapping relationships among cultivars based on their metabolomic signatures.

## 2 Results and Discussion

### 2.1 Mapping Hops' Diversity

Hierarchical cluster analysis (HCA) enabled an exploratory interpretation of the results in a graphical and intuitive manner. The resulting dendrogram revealed two main groups (Figure 1), where group 1 was mostly composed of hop cultivars from North American lineage, and group 2 was composed of hop cultivars from European lineage. This clustering pattern suggests that, regardless of the geographical origin of the hops, cultivars from related ancestral lineages tend to share similarities in their chemical profiles.

**Figure 1:**
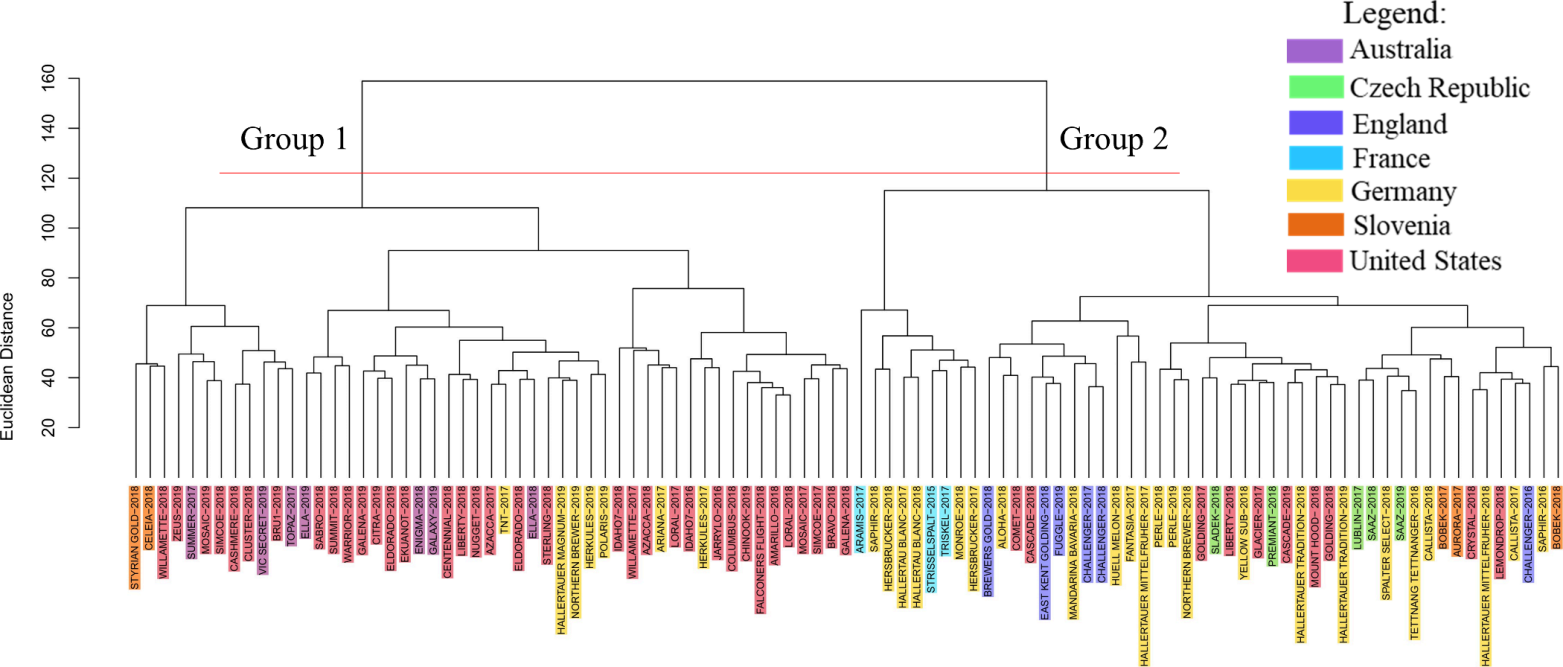
Hierarchical cluster analysis (HCA) was obtained for hop cultivars from different geographical origins. The hop names-harvest year have been color-coded according to their origin.

Principal component analysis (PCA) (Figure S1, Supporting Information) confirmed the clustering tendency observed in the HCA, where most of the hop cultivars originating from American lineage were clustered in the lower region of the score plot while hop cultivars from European lineage were clustered in the upper part of the plot.

Molecular phylogeny studies involving wild hops from different geographical origins revealed a strong genetic relationship between North American and Asian wild hops, distinguishing them from European hops.^4,34–36^ The prevalence of North American cultivars in group 1 (Figure 2) corroborates those findings. A study involving hop genetic markers revealed that native North American hops possess a greater number of unique alleles compared to European hops, indicating higher genetic diversity within North American populations.^36^ This phenomenon may result from multiple migrations of heterozygous populations to North America over time, leading to the establishment of distinct genetic profiles in the region.^4^ Given that genetics is a key factor influencing the chemical composition of hops,^34,37–39^ the two main groups observed in the dendrogram reflect metabolomic differences in hops based on their geographical lineage (i.e., hop cultivars originated from North American lineage versus hop cultivars from European lineage).

**Figure 2:**
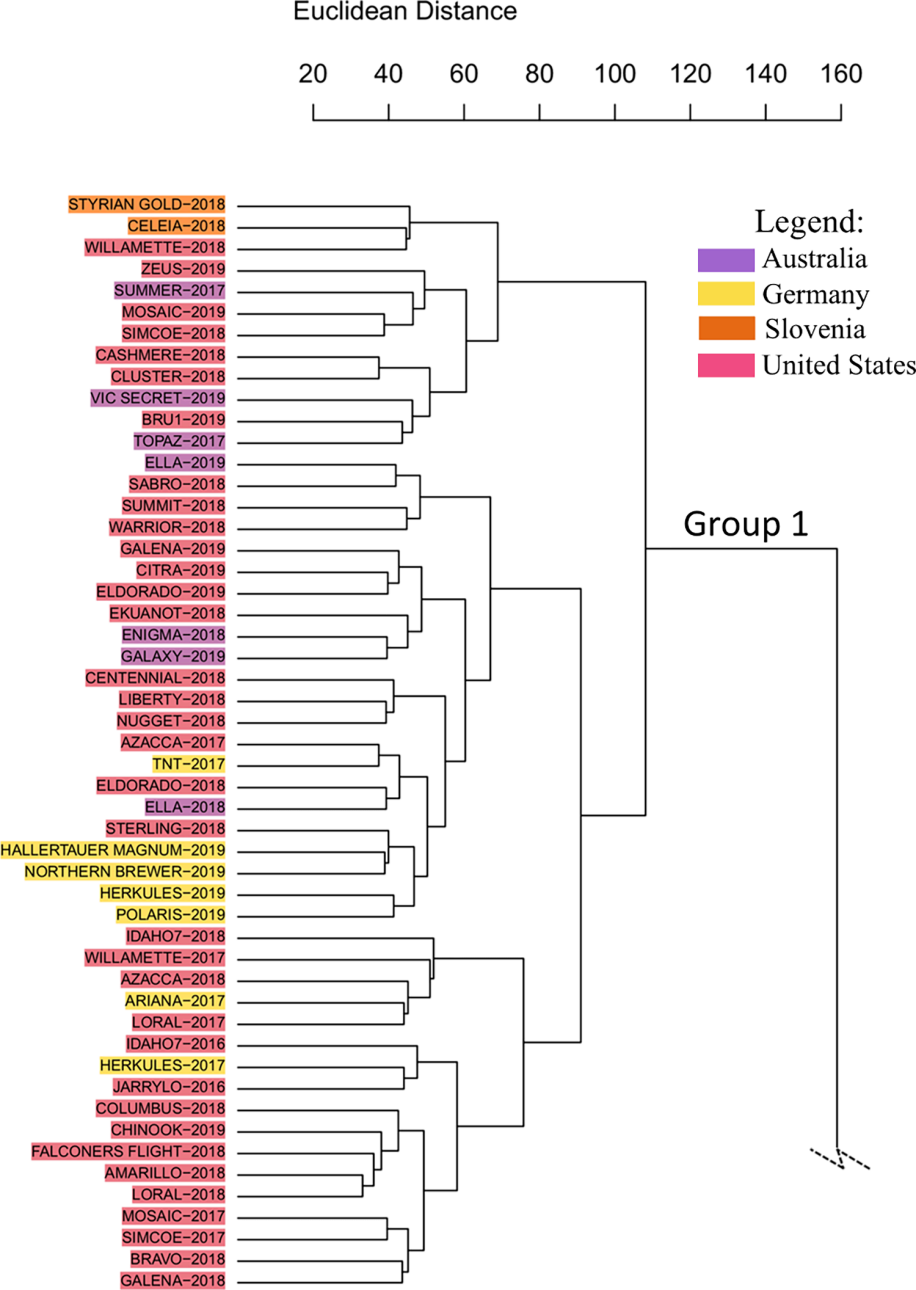
Section of the HCA dendrogram obtained for hop cultivars from different geographical origins, showing Group 1 composed mostly of cultivars with ancestral North American lineage. The hop names-harvest year have been color-coded according to their origin.

The greater genetic variability found in North American hops was a key factor driving the use of these populations in breeding programs, combining high α-acid native American hops with aromatic European hops.^40,41^ In addition to their ability to accumulate a high content of resins (12–18% of α-acids) that are responsible for adding value to the product, American hops are also known for their complex aromatic characteristics, often described as fruity, citrusy, and floral.^42^ American lineage cultivars, such as Citra, Columbus, Zeus, Chinook, Warrior, Amarillo, Azzaca, Bravo, Sabro, and Galena and their relatives Nugget, Mosaic, and Simcoe, were all clustered in group 1 of the dendrogram (Figures 1 and 2). Due to their high content in both bitter and aromatic compounds, these cultivars are among the most traded hops on the world market.^43^

In addition to hop cultivars from American lineage, the first group of the dendrogram was also composed of some hop cultivars originating from European lineage, including the German cultivars TNT, Hallertauer Magnum, Herkules, Polaris, and Ariana. These are modern European cultivars often described as having fruity and sweet aromatic notes, which resemble the aromatic profile of most American hops.^42^ Additionally, these cultivars also exhibit excellent agronomical qualities in terms of resistance to diseases and pests.^1,38^ Certain genetic relationships may be correlated to the chemical similarities observed between some cultivars in the dendrogram, such as the Hallertauer Magnum cultivar, which carries North American germplasm (a direct descendant of the American cultivar Galena), and the Ariana cultivar, which descends from the Herkules cultivar.^34,42^

Interestingly, the HCA grouped the Australian hop cultivars into an American lineage group. Ella, Enigma, Galaxy, Topaz, Vic Secret, and Summer are cultivars developed by Hops Products Australia (HPA) and successfully reflect the outcomes of the country’s hop breeding program.^44^ Many Australian cultivars contain European hop germplasm, such as Enigma (descended from the German cultivar Tettnang Tettnanger), Galaxy (with 25% ancestry from the German cultivar Perle), Summer (descended from the Czech Saaz hop), and Topaz and Vic Secret (offspring of Wye College genotypes, which are derived from a blend of English, European, and North American hops).^42,44^ Nevertheless, all of these cultivars genetically differ from traditional European hops since they carry Australian breeding genetics. Additionally, the harsh edaphoclimatic conditions in Australia are associated with unique plant adaptations, which in turn impact the chemical composition of hops grown in the country. As a result, Australian hops are categorized as high α-acid cultivars with complex and distinctive aromas.^45–47^

A particular arrangement was observed for Slovenian hops, where Styrian Gold and Celeia cultivars were grouped together in group 1 of the dendrogram (Figure 2), while Aurora and Bobek cultivars were placed in group 2 (Figure 3). The proximity of the triploid cultivars Celeia and Styrian Gold highlights their genetic relationship, as both are direct descendants of Savinjski Golding hop (a traditional Slovenian hop originated from English Fuggle hop).^48^ In addition, Celeia is known for contributing aromas and flavors reminiscent of citrus notes to beers, a characteristic of several hop cultivars that were clustered in group 1 of the HCA.^42,48^ On the other hand, the Aurora and Bobek cultivars, both in group 2 of the dendrogram, also belong to the same lineage (developed from the German hop Norther Brewer) and carry European and Slovenian genetic material.^49^

**Figure 3:**
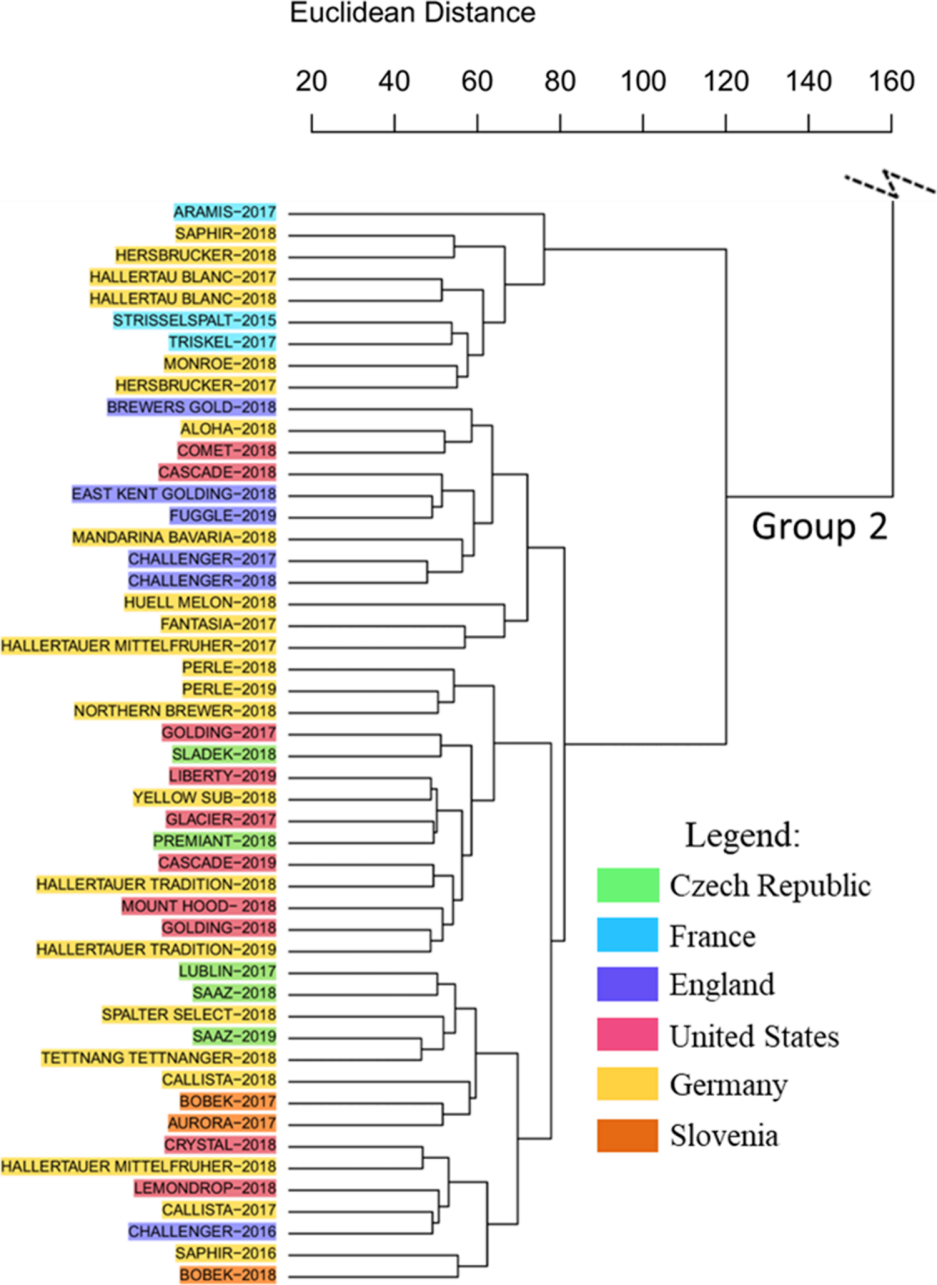
Section of the HCA dendrogram obtained for hop cultivars from different geographical origins, showing Group 2 composed mostly of cultivars with ancestral European lineage. The hop names-harvest year have been color-coded according to their origin.

The second group of the HCA mainly consisted of European hops, including varieties originated from Germany, the Czech Republic, England, France, and Slovenia, along with a few North American hops. The formation of a group containing hops from multiple European regions is reasonable, given that native wild European hops have historically been dispersed through human activity.^9^

Compared with North America, the hop selection process in Europe took place over a much longer period. Limitations in the genetic variation between European cultivars indicate that the creation of new cultivars started from a restricted number of plants native to the continent.^50^ The European cultivars Saaz, Tettnang Tettnanger, Hallertau Mittelfrueh, and Spalter, traditionally known for having an aroma profile described as noble, have been widely used in breeding programs to develop cultivars throughout history.^4,6^

Among Czech hops, the Saaz cultivar (named after its origin in Žatec/Saaz in the Czech Republic) represents one of the main European noble hops with fine aromas.^51,52^ Saaz has been the most widely cultivated hop in the Czech Republic since the Middle Ages. As it preserves the original European germplasm, it is considered the foundation for the genetic improvement of aromatic hops in the country.^52,53^ The Lublin cultivar is a direct descendant of the Saaz hop, while the Premiant and Sladek cultivars were developed from cross breeding between Saaz and Northern Brewer. Interestingly, we found that these hop varieties were all clustered together in a subgroup of group 2 of the dendrogram.

While a few German hops were assigned to group 1 of the HCA, most of the German cultivars analyzed clustered in group 2 along with their descendants and other hop cultivars from European genetic origin. Germany has one of the oldest traditions of organized hop cultivation, especially in the Hallertau, Tettnang, and Spalt regions, representing a major power in world cultivation.^54^ The hop research center, formalized in Hüll in 1926, initiated modern breeding programs focused on the research and development of new cultivars.^55^ Initially, the main objectives of the programs were to preserve the aromatic characteristics of European noble hops, as well as improve climatic adaptability and resistance to diseases (e.g., downy mildew and powdery mildew).^3^ Thus, many German hops share a genetic structure similar to that of the original European landraces, which corroborate the results observed in the HCA in which most of the German cultivars are placed in the group dominated by hops of European genetic origin (group 2).^56^

Additionally, we observed that group 2 of the dendrogram was divided into two subgroups, one of which contained the French cultivars Strisselspalt, Aramis, and Triskel. This subgroup also includes German cultivars from the Hallertau region, such as Blanc, Saphir, Hersbrucker, and Monroe. Interestingly, the French hop Strisselspalt was developed in Germany from the Hersbrucker cultivar and was used in the creation of the French cultivars Aramis and Triskel in order to guarantee the aromatic profile considered “fine” and better tolerance to diseases.^57,58^

English hops, which were allocated to group 2 in the dendrogram, are widely recognized for their classic aromas, which include earthy, spicy, floral, and woody notes.^3,42^ These hops play a fundamental role in the history of brewing, especially in traditional English beer styles.^14^ English hops have been used in many breeding programs aimed at developing new cultivars, where their influence is reflected not only in aromatic characteristics, but mainly in greater resistance to disease.^40,54^ Fuggle hops (named after their grower Richard Fuggle) were established over 150 years ago and, like East Kent Golding (named after their region of origin in Kent, England, and the Golding family responsible for their propagation), are popular hops for cultivation and beer production and exemplify the traditional European system of empirical hop propagation selection that predominated until the beginning of the 20th century.^41,54^ Challenger and Brewers Gold represent a later generation, developed in the breeding program at Wye College in England, which sought to improve disease resistance and adapt varieties to industrial needs.^19,38,59^ All of the English cultivars mentioned carry the germplasm of native Continental European hops, which aligns with the observed clustering trend.

The American cultivars Cascade, Comet, Golding, Liberty, Glacier, Mount Hood, Crystal, and Lemondrop, clustered in group 2, have European genetic descent, which may explain such grouping tendency. The Cascade cultivar, for example, was developed from the English cultivar Fuggle through the breeding program of the United States Department of Agriculture (USDA) at Oregon State University and was released in 1972 as an aromatic cultivar.^42,60^ The Lemondrop cultivar also retains genotypic characteristics of European hops since it was created from Cascade. The triploid cultivars Crystal and Mount Hood were developed from the German aromatic cultivar Hallertauer Mittelfrüher, and Crystal also received contributions from the American Cascade and the English Brewer’s Gold hops.^38,42^

The cultivars Crystal, Mount Hood, and Liberty are considered siblings due to their similar relationships and chemical profiles.^42^ The Golding cultivar was created from the English hop Brewer’s Gold, while the Glacier cultivar, a descendant of the French Strisselspalt, also carries contributions from Northern Brewer and Brewer’s Gold. The Comet cultivar, developed by the USDA breeding program and registered in 1962, is a high α-acid cultivar that is also descended from both English and native American hops.^61^ These examples highlight the genetic complexity of modern hop cultivars resulting from the hybridization of European and American hops.

Some inconsistencies in clustering due to the harvest year were noted, such as the Challenger sample (2016 harvest, group 2), Liberty (2018 harvest, group 1), and Northern Brewer (2019 harvest, group 1). While our results demonstrate that genetic lineage remains the primary driver of chemical clustering, various factors are known to directly influence hop chemical composition and may contribute to the observed inconsistencies. The chemical composition of hops is highly sensitive to environmental variables such as climatic conditions (temperature, rainfall, and solar radiation), soil composition, geographical location, and agricultural management practices, which can affect the biosynthesis and accumulation of secondary metabolites.^28,45,46,62^ Postharvest factors including processing methods, drying conditions, storage environment, packaging, and transportation logistics also significantly alter the chemical profile by affecting compound stability and degradation.^63^ Additionally, factors such as harvest timing and plant maturity contribute to the complexity of hop chemical composition by influencing metabolite concentration and maturation.^64^ Nevertheless, the overall consistency of clustering patterns across most samples from different harvest years supports the evidence that genetic factors provide a robust framework for hop classification.

### 2.2 Exploring the Hop Chemical Content

To explore variations in the chemical profiles of hops based on the clustering patterns observed in the HCA, a molecular network was generated using the same GC-MS data (Figure S2, Supporting Information). Each node in the network represents a detected metabolite, which are arranged into clusters according to their MS spectral similarities. This approach enabled the annotation of key metabolites responsible for differentiation between the two main groups observed in the dendrogram.

The clusters containing the major annotated metabolites are shown in Figure 4. The largest cluster is primarily composed of sesquiterpenoids (dark blue border), one diterpenoid (pink border), and several unidentified metabolites. Other clusters are composed of monoterpenoids (light blue border), aliphatic esters (green border), ketones (brown border), and tocopherols (yellow border).

**Figure 4:**
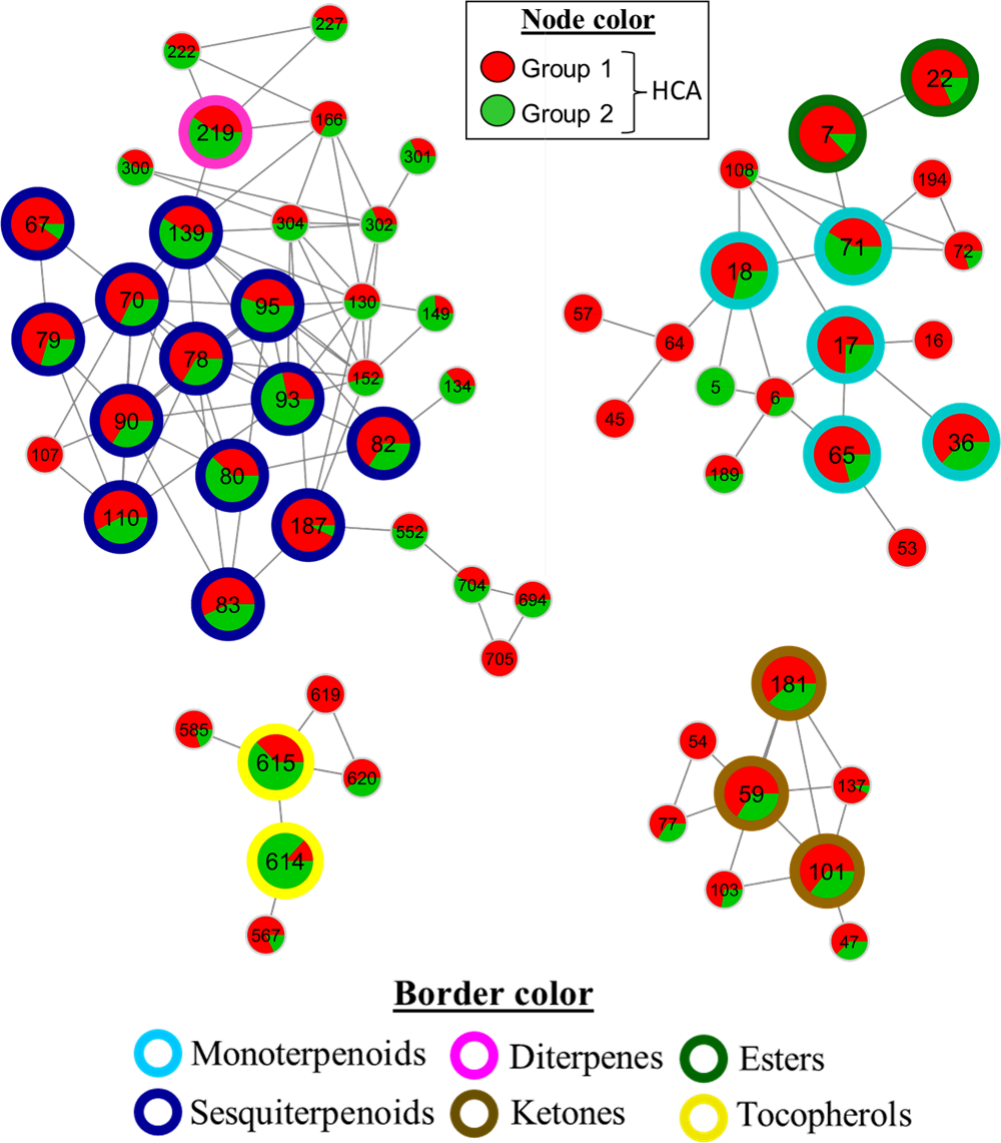
Clusters containing annotated metabolites selected from the molecular network. The numerical labels inside the nodes indicate the respective identification numbers (node IDs). The nodes are colored according to the groups obtained from the unsupervised hierarchical cluster analysis (HCA), and the border colors correspond to the metabolite classes.

Twenty-six metabolites were annotated in level 2 of confidence according to the minimum reporting standards for chemical analysis (Table 1).^65,66^ The group 1 of the HCA dendrogram, composed predominantly of North American hops and their descendants, was characterized by a higher abundance of monoterpenoids, sesquiterpenoids, aliphatic esters, and methyl ketones, annotated as β-myrcene (**17**), 2,6-octadiene, 2,7-dimethyl (**18**), *trans*-geranic acid, methyl ester (**65**), linalool (**36**), calarene (**79**), α-cubebene (**67**), α-copaene (**70**), β-caryophyllene (**78**), α-humulene (**82**), β-farnesene (**83**), germacrene D (**90**), γ-cadinene (**110**), farnesol (**187**), isobutyl isobutyrate (**7**), 2-methylbutyl isobutyrate (**22**), 2-undecanone (**59**), 2-tridecanone (**101**), and 2-pentadecanone (**181**). The group 2, consisting of European hops and their descendants, was distinguished by higher prevalence of the monoterpenoid geranyl acetate (**71**), the sesquiterpenoids α- and β-selinene (**95** and **93**), *trans-*α-bergamotene (**80**), humulene epoxide II (**139**), the diterpenoid neophytadiene (**219**), and β- and γ-tocopherols (**614** and **615**).

**Table 1:** Major Metabolites Annotated in the Molecular Network Generated from GC-MS Data^a^

node ID	compound name	Rt (min)	Ri calc	Ri lit	class
18	2,6-octadiene, 2,7-dimethyl	5.64	994	985	monoterpenoids
17	β*-*myrcene	5.61	992	992	
36	linalool	8.65	1105	1103	
65	*trans*-geranic acid, methyl ester	15.73	1332	1324	
71	geranyl acetate	17.53	1389	1381	
67	α-cubebene	16.34	1352	1351	sesquiterpenoids
70	α-copaene	17.17	1378	1375	
78	β-caryophyllene	18.52	1421	1418	
79	calarene	18.83	1432	1432	
80	*trans-*α-bergamotene	19.02	1438	1438	
82	α-humulene	19.56	1456	1454	
83	β-farnesene	19.6	1457	1454	
90	germacrene D	20.27	1479	1480	
93	β-selinene	20.58	1490	1488	
95	α-selinene	20.85	1498	1494	
110	γ-cadinene	21.41	1518	1513	
139	humulene epoxide II	24.22	1615	1606	
187	farnesol	27.38	1730	1722	
219	neophytadiene	30.2	1812	1806	diterpene
7	isobutyl isobutyrate	4.05	914	913	aliphatic esters
22	2-methylbutyl isobutyrate	6.22	1017	1016	
59	2- undecanone	14.88	1305	1294	ketones
101	2-tridecanone	20.97	1503	1491	
181	2-pentadecanone	26.69	1704	1699	
614	β-tocopherol	54.31	3057	3043	tocopherols
615	γ-tocopherol	54.37	3061	3055	

a Node ID, identification number of the node in the molecular network; Rt (min), retention time in minutes; Ri calc, calculated retention index; Ri lit, retention index from the literature (https://webbook.nist.gov/chemistry/name-ser/).

Group 1 hop cultivars were mainly characterized by the presence of the sesquiterpenoids α-humulene and β-caryophyllene, as well as the monoterpene β-myrcene. These three compounds are recognized as the main constituents of hop essential oils and account together for up to 90% of the total composition, depending on the cultivar.^2^ The predominance of β-myrcene in North American hops, consistently reported in the literature, was confirmed in this study.^67^ Alongside β-myrcene, a higher concentration of linalool was also observed, a monoterpenoid with floral aromatic properties that shares the same biosynthetic pathway as β-myrcene,^68^ which explains their co-occurrence and prominence in the North American lineage group.

In addition to this chemical profile, the presence of several other sesquiterpenoids (calarene, α-cubebene, α-copaene, germacrene D, γ-cadinene, and farnesol) was also observed in group 1 hops. The biosynthesis of sesquiterpenes generates a wide diversity of chemical structures, which are primarily modulated by genetic factors through the action of specific sesquiterpene synthase enzymes.^69^ The biosynthetic transformation pathway of this metabolite class may explain the simultaneous and abundant presence of these compounds within the same group, corresponding to hops of North American lineage.

Also, group 1 cultivars exhibited high levels of β-farnesene, a sesquiterpene initially isolated from the Czech hop Saaz and traditionally reported as a chemical marker of European hops.^67,70^ Despite this long-standing association, the presence of this metabolite is highly variable among hop cultivars, and its absence has been documented in several traditional European varieties such as Hallertau, Goldings, and Fuggles.^19^ Our results, comprising 76 different hop cultivars, revealed a high predominance of β-farnesene in the group of North American hops. Nevertheless, the results are consistent with previous research demonstrating an inverse relationship between farnesene and selinene levels in hops. These findings were also observed in our study, considering that α- and β-selinenes were predominantly detected in the European hop group.^67^ Interestingly, selinenes have been described as effective chemical markers for the identification of hop cultivars, with several studies reporting high concentrations of these compounds in hops of European genetic background.^41,51,67,70–72^ Thus, our findings reinforce the evidence that selinenes are potential metabolites for distinguishing hops from European lineages.

Still within the class of sesquiterpenes, *trans-*α-bergamotene was among the most prominent annotated metabolites in the group of hops from the European lineage. This compound is typically a minor sesquiterpene in hop essential oils, yet it can contribute floral and citrus-like aromatic characteristics to the hops.^46^ In a study aimed at differentiating 12 hop cultivars through essential oil analysis, including both European and non-European varieties, *trans-*α-bergamotene was identified as a key marker for the European cultivars Saaz, Lublin, and Styrian.^73^ In our previous study comparing the cultivars Chinook, Nugget, Centennial, and Columbus grown in different regions (in the USA and Brazil), *trans-*α-bergamotene demonstrated high discriminative power, as it was detected in the samples cultivated in Brazil and absent in those cultivated in the United States.^74^ The presence of this metabolite has been reported only in specific cultivars,^75^ and the data collected support its potential as a chemical marker associated with hop origin. Consistently, our results revealed a similar pattern, with a low occurrence of this compound in North American hops, further reinforcing its identification as a biomarker metabolite within the European hops group.

In addition to a sesquiterpene-rich profile, the group of North American hops and their descendants also stood out for the presence of oxygenated compounds, such as the ketones 2-undecanone, 2-tridecanone, and 2-pentadecanone, as well as the aliphatic esters isobutyl isobutyrate and 2-methylbutyl isobutyrate. Methyl ketones are especially relevant because they contribute to floral and fruity sensory notes to hops.^2^ The first and most abundant ketone identified in hop oils,^76^ 2-undecanone, has been described as a promising chemical marker for differentiating cultivars.^73,77^ In this study, we found that 2-undecanone was detected in greater abundance in North American hops than in European lineage hops.

The identified branched-chain esters, isobutyl isobutyrate and 2-methylbutyl isobutyrate, derive from metabolic pathways associated with amino acid biosynthesis.^19^ The presence of isobutyric esters has been reported in relatively higher concentrations in modern hops with high α-acid content, while they occur at significantly lower levels in traditional European hops.^78^ This characteristic aligns with our results, where isobutyl isobutyrate and 2-methylbutyl isobutyrate were identified as discriminant metabolites for North American lineage cultivars.

Oxygenated compounds represent the group of hop metabolites with the greatest chemical complexity, although they are present in lower proportions in the total composition of hop oils (up to 30%).^76^ These compounds generally have low sensory perception thresholds and, therefore, can exert a significant influence on the aromatic characteristics of hops, often associated with floral and fruity notes.^2,19^ The main aromatic oxygenated compounds highlighted in the group of hops with European lineage were geranyl acetate and humulene epoxide II. The latter is an oxidation product of the sesquiterpene α-humulene and tends to be predominant among the oxidized derivatives, with increasing concentration during hop storage. Epoxides, in general, are recognized as important contributors to the aromatic profile of noble hops, associated with herbaceous and spicy notes typical of many European cultivars that were allocated to group 2 of the HCA dendrogram in our analysis.^68,79^ In turn, the terpene ester geranyl acetate, derived from the monoterpenoid geraniol and also highlighted in group 2 hops, contributes floral notes to its sensory profile.^19,78^

The presence of tocopherols, especially β- and γ-tocopherol forms, was also highlighted in the group of European hops. Tocopherols are lipophilic metabolites widely recognized for their significant antioxidant activity.^80^ In addition to hops, tocopherols are also present in barley malt, and when present in appropriate amounts, they contribute as agents that promote the organoleptic stability of beers.^19,81^

## 3 Conclusions

Our metabolomic approach of hops cultivars based on GC-MS analyses and unsupervised statistical methods demonstrated a consistent correlation with their genetic origin (North American and European lineages). Molecular networking analysis enabled the annotation of 26 metabolites that contributed to differentiating North American from European lineage hop cultivars. Overall, hops of North American lineage exhibited chemical profiles characterized by mono- and sesquiterpenoids, ketones, and esters. Furthermore, the results reinforced the evidence supporting selinenes as relevant markers for European-derived hops and highlighted *trans-*α-bergamotene as an important chemical marker. This metabolomic technique proved to be an effective strategy for mapping relationships among hops based on their chemical profiles and provided a powerful and comprehensive scientific foundation for the characterization of different hop cultivars commercially available to the brewing industry.

## 4 Methods

### 4.1 Acquisition of Hop Samples

A total of 76 different commercial hop cultivars were acquired for this study, including hops from different harvest years, thus resulting in 101 samples. Only hops provided in modified atmosphere packaging and in a pelletized form were utilized, as pelletized hops represent the most common commercial form used in brewing. Information regarding the cultivar names, harvest year, and origin is provided in Table S1, Supporting Information.

### 4.2 Extract Preparation and GC-MS Analysis

The methodologies used for extract preparation and the GC-MS analytical conditions were the same as those previously employed by our research group.^74^ Blank samples (dichloromethane) and hydrocarbon standard solution (C_8_–C_40_, Supelco, Sigma-Aldrich, St. Louis, MO, USA) were also injected during the chromatographic analysis.

Analyses were conducted using a gas chromatograph coupled to a quadrupole mass spectrometer (QP2010 Ultra, Shimadzu Corporation, Kyoto, Japan). The chromatograms and mass spectra were visualized using GC Solutions software (version 4.20 for Windows, Shimadzu Corp., Kyoto, Japan).

### 4.3 GC-MS Data Processing

The data generated from the analyses were processed using MZmine software (version 2.53 for Windows, BMC Bioinformatics, UK) with the ADAP algorithm sequence for GC-MS.^82^ To this end, the data were converted into a *.mzXML file format using GC Solutions software and then loaded into the software.

The parameters used for the mass detection, baseline correction, chromatogram construction, deconvolution, and peak alignment stages were as follows: mass detection, mass detector-centroid (noise level, 5 × 10^2^); ADAP chromatogram builder (min group size in # of scans, 5; group intensity threshold, 5 × 10^2^; min highest intensity, 1 × 10^3^; *m*/*z* tolerance, 0.5 *m*/*z* or 0 ppm); chromatogram deconvolution, algorithm-wavelets (ADAP) (S/N threshold, 10; S/N estimator, intensity window SN; min feature height, 1 × 10^3^; coefficient/area threshold, 100; peak duration range, 0.02–2.0; RT wavelet range, 0.01–0.20), *m*/*z* center calculation–median; hierarchical clustering (min cluster distance (min), 0.01; min cluster size, 3; min cluster intensity, 1 × 10^3^; min edge-to-height ratio, 0.3; min delta-to-height ratio, 0.2; min sharpness, 90; shape-similarity tolerance, 70; choice of model peak based on, *m*/*z* value); alignment, ADAP aligner (GC) (min confidence, 0.01; retention time tolerance, 0.3 min (absolute); *m*/*z* tolerance, 0.5 *m*/*z* or 0 ppm; score threshold, 0.7 e retention time similarity, retention time difference, 0.4). The processed data were saved in spreadsheets with comma-separated values (CSV*)* and Mascot generic format (MGF) for later data analysis.

### 4.4 Statistical Analysis and Molecular Network

The data obtained after processing were used in correlation analyses through multiple statistical treatments. The *.csv matrix containing the quantitative data was transformed to a logarithmic scale (Log10) and subjected to unsupervised hierarchical cluster analysis (HCA) using R software (R Project for Statistical Computing). The Ordinary Euclidean Distance algorithm and Ward’s linkage method were used. In addition, PCA was performed using SIMCA software (version 13.0.3.0 for Windows, Umetrics, Umeå, Sweden), including data from the blank samples and hydrocarbon standards (C_8_–C_40_). The respective clustering of the blank samples and the hydrocarbon standard samples confirmed the analytical reproducibility and the suitability of the data processing approach (Figure S1, Supporting Information).

A molecular network was created using the Library Search/Molecular Networking GC workflow on the Global Natural Products Social Molecular Networking (GNPS) platform (https://gnps.ucsd.edu/ProteoSAFe/static/gnps-splash.jsp?redirect=auth).^83^ The following parameters were applied: fragment ion mass tolerance, 0.5 Da; min matched peaks, 6; score threshold, 0.8; library class, bronze; top hits per spectrum, 10; filter StdDev intensity, 0; filter SNR intensity, 0; min peak int, 0; filter precursor window, do not filter; filter library, do not filter; filter peaks in a 50 Da window, do not filter; min pairs cos, 0.6; network topK, 10; maximum connected component size, 50; perform Kovats calculation, on; precursor ion mass tolerance, 20,000 Da; search analogs, do not search; maximum analog search mass difference, 100; maximum shift between precursors, 500; force exact match, 1. The molecular network was visualized and analyzed using Cytoscape software (version 3.7.2 for Windows, Institute for Systems Biology, Seattle, WA, USA).

The annotation of the metabolites was based on the molecular network obtained in the GNPS platform, the comparison of the mass spectra with data libraries (NIST 11s, FFNSC 1.3, and WILEY 7 libraries), and the comparison of the retention indices found in the literature with those calculated according to van Den Dool and Kratz.^84^

## Supplementary Material





## Notes

(Figure S1) Principal component analysis (PCA) score plot based on the first and second components; (Figure S2) molecular network generated from the data obtained from the GC-MS analysis (PDF)
(Table S1) Information regarding the cultivar names, harvest year, origin, and breeding/ancestry (XLSX)
