# Supplementary material for: Mapping Metabolomic Relationships of Hop Cultivars in an Ancestral Lineage Context
Source: ACS Omega. 2025 Sep 29;10(40):47281–91. doi: 10.1021/acsomega.5c06162 (PMC12529167; doi:10.1021/acsomega.5c06162)
Supplement: Supplementary file 1 [file ao5c06162_si_001.pdf]

Mapping metabolomic relationships of hop cultivars in an ancestral lineage context

Guilherme Silva Dias[1], *Marília Elias Gallon*[1] and *Leonardo Gobbo-Neto*[1]*

1. Department of BioMolecular Sciences, School of Pharmaceutical Sciences of Ribeirão Preto, University of São Paulo (USP), Av. do Café s/n◦, Ribeirão Preto, SP, 14040-903, Brazil.

*Correspondence: gobbo@fcfrp.usp.br

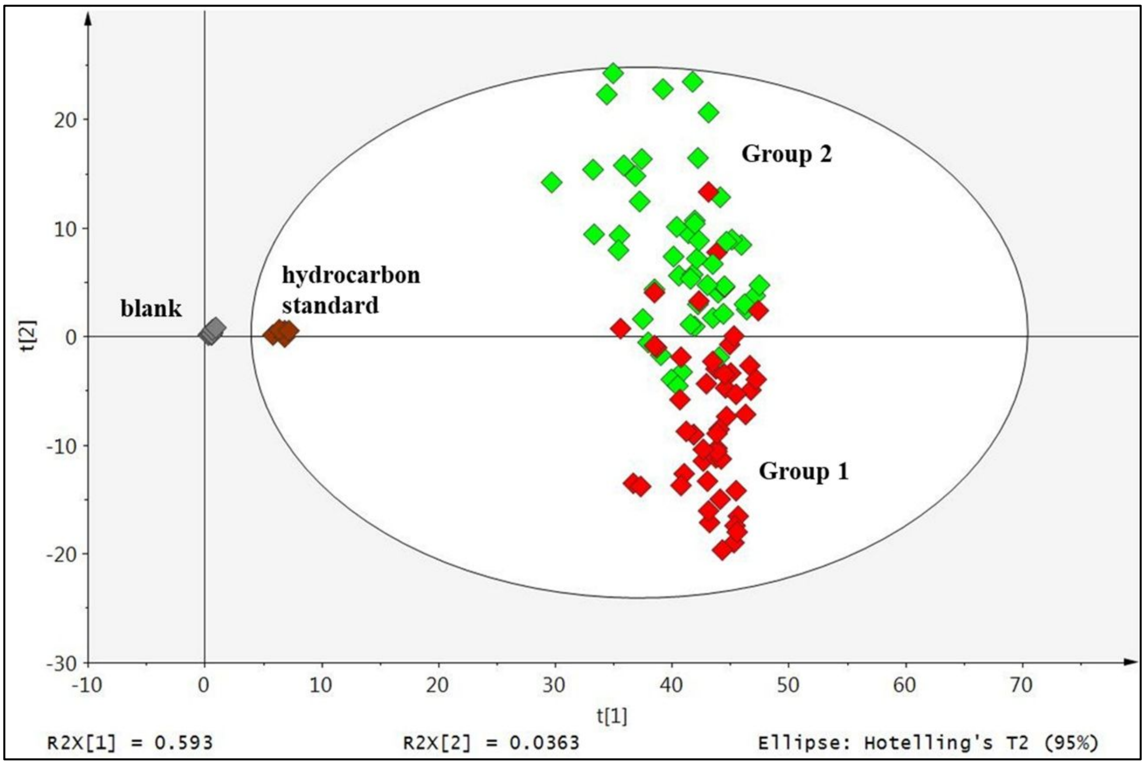

Figure S1. PCA score plot based on the first and second principal components explaining 59.3% and 3.63% of the data variance, respectively (R^2X = 0.767; Q^2 = 0.591). The samples are represented as colored diamonds (grey, blank; brown, hydrocarbon pattern; red and green, Group 1 and Group 2 as observed in the HCA analysis, respectively). R^2X, goodness of fit for the X variables, Q^2, model robustness.

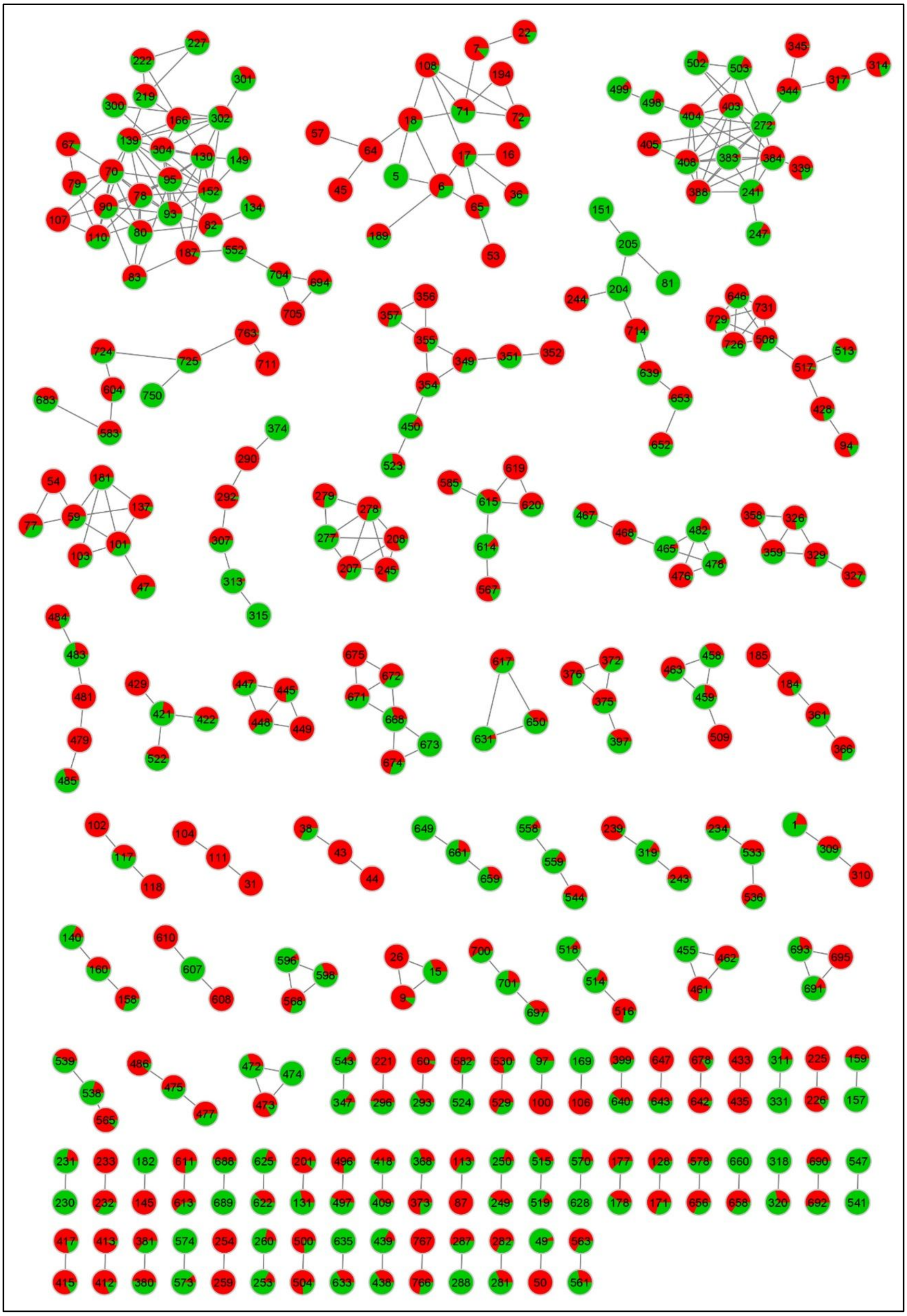

Figure S2. GC-MS-based molecular networking analysis for commercial hop cultivars. The nodes are colored according to the groups obtained in HCA analysis. (red, American lineage hops; green, European lineage hops). The numerical labels inside the nodes indicate the respective identification of the node (Node ID).
